# Screening of different growth conditions of *Bacillus subtilis* isolated from membrane-less microbial fuel cell toward antimicrobial activity profiling

**DOI:** 10.1515/biol-2022-0809

**Published:** 2024-01-24

**Authors:** Dharni Kuhan Sreedharan, Hartini Alias, Muaz Mohd Zaini Makhtar, Tan Joo Shun, Ana Masara Ahmad Mokhtar, Hafiza Shukor, Masoom Raza Siddiqui, Mahboob Alam, Riti Thapar Kapoor, Mohd Rafatullah

**Affiliations:** Bioprocess Technology Division, School of Industrial Technology, Universiti Sains Malaysia, Penang 11800, Malaysia; Centre for Innovation and Consultation, Universiti Sains Malaysia, 11800 Penang, Malaysia; Renewable Biomass Transformation Cluster, School of Industrial Technology, Universiti Sains Malaysia, Penang 11800, Malaysia; Centre of Excellence for Biomass Utilization, Faculty of Chemical Engineering Technology, Universiti Malaysia Perlis, 02600 Arau, Perlis, Malaysia; Chemistry Department, College of Science, King Saud University, Riyadh 11451, Saudi Arabia; Division of Chemistry and Biotechnology, Dongguk University, 123, Dongdaero, Gyeongju-si, 780714, Republic of Korea; Centre for Plant and Environmental Biotechnology, Amity Institute of Biotechnology, Amity University Uttar Pradesh, Noida, 201 313, Uttar Pradesh, India; Environmental Technology Division, School of Industrial Technology, Universiti Sains Malaysia, Penang 11800, Malaysia

**Keywords:** microbial fuel cells, membrane, energy, wastewater, treatment.

## Abstract

Bacteriocins produced by *Bacillus subtilis* have gained recognition for their safe use in humans. In this study, we aimed to assess the inhibitory activity of an antimicrobial peptide synthesized by the wild-type strain of *B. subtilis* against the notorious pathogen *Pseudomonas aeruginosa*. Our investigation employed the broth microdilution method to evaluate the inhibitory potential of this peptide. Among the four different pathogen strains tested, *P. aeruginosa* exhibited the highest susceptibility, with an inhibition rate of 29.62%. In parallel, we explored the cultivation conditions of *B. subtilis*, recognizing the potential of this versatile bacterium for applications beyond antimicrobial production. The highest inhibitory activity was achieved at pH 8, with an inhibition rate of 20.18%, indicating the potential for optimizing pH conditions for enhanced antimicrobial peptide production. For the kinetics of peptide production, the study explored different incubation periods and agitation levels. Remarkably, the highest activity of *B. subtilis* was observed at 24 h of incubation, with an inhibition rate of 44.93%. Finally, the study focused on the isolation of the antimicrobial peptide from the cell-free supernatant of *B. subtilis* using ammonium sulfate precipitation at various concentrations. The highest recorded activity was an impressive 89.72% achieved at an 80% concentration.

## Graphical abstract

**Figure:**
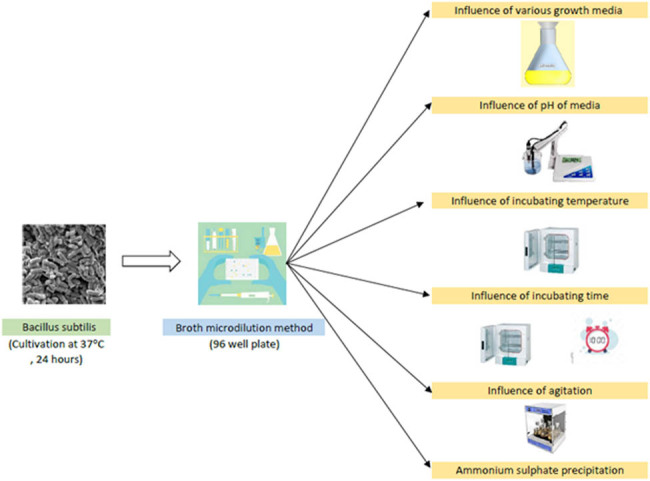

## 1 Introduction

In this current age, demand for antibiotics is accelerating at an alarming rate, especially for the treatment of human-related disorders and growth facilitators, as well as in livestock husbandry. Antibiotics have been conventionally used for the treatment of human and animal infections. However, due to their adverse impact on the environment, these compounds are regarded as emerging contaminants [1,2]. Antibiotics consumed by both humans and animals do not experience any degradation during excretion [3,4]. Moreover, conventional wastewater treatment methods fail to effectively remove these compounds, which stay adhered to sludge. The activated sludge technique is currently used mostly by wastewater treatment facilities to meet the goals of denitrification and dephosphorization, although the antibiotics elimination rate is relatively poor [5].

This research shows that sludge is the main source of fluoroquinolones, and the application of biosolids to agricultural regions has the potential to discharge these compounds into the environment [6]. The presence of antibiotics, even in trace amounts, can promote the development of multidrug resistance genes in bacteria, acting as a selective pressure for microbial acclimation. As a result, the chances of a microorganism gaining such exposure and surviving are likely to promote the horizontal transfer of genes between the microbes. *Pseudomonas aeruginosa* is one such pathogen that can be found in wastewater effluent, and due to its innate resistance to various antibiotics, *P. aeruginosa* is able to withstand and survive in wastewater for an extended period of time [7,8].

Microbial fuel cell (MFC), a green technology, degrades organic compounds in wastewater using microorganisms as biocatalysts, converting chemical energy to bioelectrical electrical energy during this process [9,10]. For the MFC, various inoculum sources (food waste, lignocellulose material, anaerobic digester, or brewery sludge) were used, with the goal of adapting electroactive community of microbes and subsequent treatment of wastewater. Electroactive bacteria, also known as “electrogens,” are bacteria that can generate electricity. *Geobacter*, *Shewanella,* and *Arcobacter* genera are recognized as electrogens, but MFCs also contain other genera such as probiotics [11].

The rapid increase in antibiotic-resistant strains has prompted the development of alternative bacterial infection therapies. Bacteriocins are a heterogeneous class of bactericidal peptides or proteins produced by bacteria. Probiotics like lactic acid bacteria (LAB) are capable of producing an antimicrobial compound known as bacteriocin to inhibit closely related strains [12,13]. Bacteriocins are small cationic molecules with hydrophobic or amphiphilic properties that exhibit inhibitory activity against closely related species. Bacteriocins consist of a miscellaneous cluster of ribosomally synthesized peptides with <60 amino acid residues. However, unlike the bacteriocins from LAB, the classification of bacteriocins generated by *Bacillus* is scanty. *Bacillus* species have been recognized to be safe for food and industry, and many bacteriocin-producing bacteria have been used as probiotics. Bacteriocins synthesized by *Bacillus subtilis* garnered the highest popularity in various applications due to their safe application in humans [14,15]. Industrially significant bacteriocin is produced by several *Bacillus* species and has attained generally recognized as safe status due to the history of their safe application [16]. Therefore, bacteriocins are gaining demand to replace the use of antibiotics. The natural bioactive peptides or bacteriocins produced from *Bacillus* species prevent food spoilage by inhibiting the activity of pathogens and are beneficial for consumer health and industrial applications.

Therefore, the present investigation was carried out to screen the suppressing action of an antimicrobial compound synthesized by a wild-type *B. subtilis* strain in a membrane-less MFC (ML-MFC) system against the locally isolated pathogen, *P. aeruginosa* ATCC 10145, and partially purify the antimicrobial compound for potential usage in bioremediation. ML-MFC commonly regarded as a green technology for renewable energy generation and bioremediation only, but without realized the biocatalyst that was used (in this study was *B. subtilis*) had another potential which having an antimicrobial properties. This contributes to a new finding that caters to energy recovery, bioremediation, and health perspectives.

## 2 Methodology

### 2.1 Cultivation and culture conditions of *B. subtilis*

The wild type of *B. subtilis* used in this study was isolated from the ML-MFC supplemented with chicken manure explored by our research team as per the procedure of Mohd Azmi et al. [17]. The isolated strain was activated again in nutrient broth (NB) for 24 h at 37°C. The overnight culture was subcultured twice before the stock culture was prepared. The stock culture was kept in 25% glycerol at −80°C (St. Louis, Missouri, USA) as per the procedure of Sharma et al. [18].

### 2.2 Antimicrobial activity screening of *B. subtilis*

The culture of *B. subtilis* was grown overnight in two different NB and tryptic soy broth (TSB) media in the ML-MFC system. Nutrient supplementation leads to power generation (as *B. subtilis* is an electrogenic bacteria that has the ability to pass electrons and current and into the system), but the study aimed for antimicrobial activity as a priority. Then, *B. subtilis* culture was centrifuged at 8,000 *g* at 4°C for 20 min. The pellet was removed, and the supernatant was filtered by applying a filter membrane (0.22 µm; Minisart^®^, Sartorius) to ensure no traces of *B. subtilis* culture in the supernatant. A few different pathogens like *Escherichia coli* 078:K80 (gram negative), *Staphylococcus aureus* (gram positive), and *P. aeruginosa* (gram negative) were applied as indicator organisms to determine the antimicrobial activity of *B. subtilis*. Pathogens were developed overnight at 37°C in NB medium and diluted to 10^6^ colony forming units. The activity was tested using broth microdilution assay in a 96-well plate. The broth microdilution method was executed as given by Sreedharan et al. [19]. The pathogen suspension (100 µL) was inoculated together with 100 µL of cell-free supernatants (CFS) in the well. De man, rogosa, sharpe (MRS) broth replaces the CFS for the negative control, while streptomycin (10 mg mL^−1^) functions as a positive control. The culture turbidity was analyzed at 596 nm after 24 h of incubation at 37°C. The following equation was used for analyses of the inhibitory action of CFS:

\[{\mathrm{Percentage\; of\; inhibition}}(I\left \% )=\frac{{\mathrm{Optical\; density\; control}}-{\mathrm{Optical\; density\; sample}}}{{\mathrm{Optical\; density\; control}}}\times 100.]\]

The absorbance of control shows changes in control (pathogens mixed with MRS broth), and the optimal density of the sample reflects alterations in sample absorbance (pathogens mixed with CFS).

### 2.3 Influence of various growth media and pH

The impact of growth media on the inhibition activity of CFS was investigated by culturing *B. subtilis* in eight different specialized growth media in the ML-MFC system. The growth media were Brain–heart infusion (BHI) broth, Luria Bertani (LB) broth, and TSB added with 1% yeast extract (TSB + YE), tryptone-yeast extract (TY) broth, NB broth, yeast malt extract (YME) broth, Mueller Hinton (MH) broth, and complex medium (Landy media [20] and nutrient medium [N 3] [21]). Both complex media were prepared accordingly, as suggested by Fuchs et al. [20] and Todorova and Kozhuharova [21]. Overnight cultivated *B. subtilis* culture was introduced into the prepared media and incubated at 37°C for 1 day. The next day, cultures were spun at 8,000 *g* for 20 min at 4°C to retrieve the CFS before being tested with a broth microdilution assay against *P. aeruginosa* to determine the antimicrobial activity [22,23]. The influence of pH on the supernatant of *B. subtilis* was studied by maintaining the pH of NB from pH 4 to pH 8 in the ML-MFC system. pH was maintained by using 1 N hydrochloric acid or sodium hydroxide. Cultured *B. subtilis* was inoculated into pH-adjusted NB and incubated at 37°C for 24 h. Then, the culture was centrifuged to harvest the CFS 8,000 *g* at 4°C for 20 min, subsequently tested its antimicrobial activity using broth microdilution assay [24].

### 2.4 Influence of incubating temperature

The impact of temperature on the inhibitory activity of *B. subtilis* was assessed by incubating at different temperatures in the ML-MFC system. Cultured *B. subtilis* was inoculated into NB and incubated at three different temperatures, i.e., 25, 30, and 45°C, for 1 day. CFS was obtained after centrifugation of culture for 20 min at 8,000 *g* and 4°C The antimicrobial activity exhibited by the CFS of *B. subtilis* was tested using broth microdilution method. The results were observed and tabulated after 24 h of incubation [25].

### 2.5 Effect of different incubation time

The influence of the incubation period was investigated by incubating *B. subtilis* for a span of 3 days in the ML-MFC system. *B. subtilis* 1% (v/v) was transferred to NB medium and incubated for 3 days at 37°C. *B. subtilis* culture was sampled at every 24 h during the 3-day period. The cultures were filtered after centrifugation for CFS, and the inhibition activity was calculated using broth microdilution assay with *P. aeruginosa* as the indicator strain [26].

### 2.6 Influence of agitation

The importance of agitation toward the inhibition activity of CFS was investigated by comparing the CFS activity from *B. subtilis* culture in the presence and absence of agitation in the ML-MFC system. *B. subtilis* (1%) culture was mixed into two separate NBs, with one culture shaken at 150 rpm while the other media was not shaken. Both cultures were placed for 24 h at 37°C. The next day, CFS of the cultures were obtained by centrifugation for 20 min at 8,000 *g*, and the inhibition activity was determined through broth microdilution assay. The inhibition activity results were observed and tabulated after 24 h of incubation [27].

### 2.7 Ammonium sulfate precipitation

The antimicrobial compound in the CFS of *B. subtilis* was extracted using the ammonium sulfate precipitation technique to determine whether the compound produced by *B. subtilis* is of proteinaceous nature. This method involves using ammonium sulfate salts to concentrate the protein present in the CFS. Different concentrations of ammonium sulfate were used from 20 to 80% w/v. *B. subtilis* culture was cultivated in 500 mL of NB with the pH adjusted to pH 5 for 1 day at 37°C. CFS was obtained by centrifuging culture at 8,000 *g* for 20 min. Upon harvesting the CFS, ammonium sulfate (20, 40, 60, and 80% w/v) was supplemented into CFS and stirred homogenously. The CFS was kept at 4°C for at least 2 h to allow the salts to congregate the protein before centrifuging it at 8,000 *g* for 15 min. Filtrate was removed, and the pellet was resuspended with 1 mL of phosphate buffered saline. The antimicrobial activity of the extracted protein was tested by broth microdilution method against *P. aeruginosa* [28].

### 2.8 Statistical analysis

Data were assessed using SPSS version 22.0 (IBM, New York, USA). The significant difference between the means was assessed by one-way ANOVA. The significance level was set at *α* = 0.05, and Tukey’s test was used for data analyses.

## 3 Results

**Figure 1:**
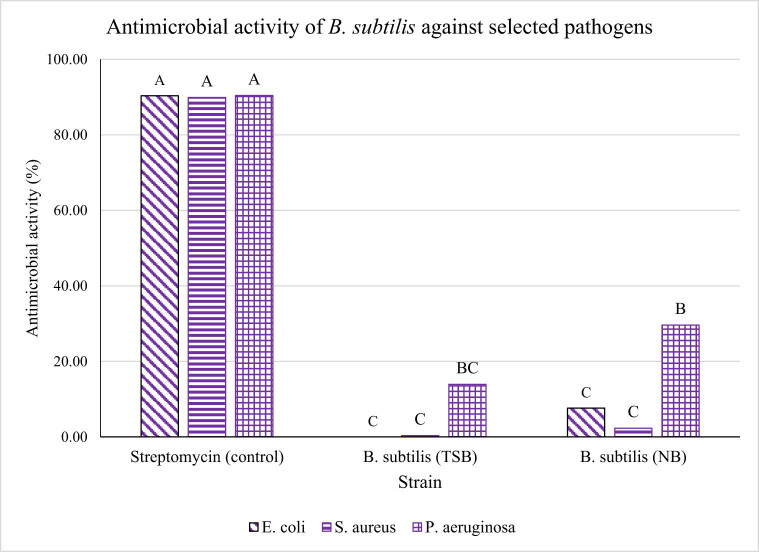
The antimicrobial activity of CFS from *B. subtilis* produced in two different media (NB and TSB). The activity was tested against three different pathogens (*E. coli* 078:K80, *S. aureus*, and *P. aeruginosa*). Streptomycin sulfate acts as a positive control. ^A,B,C^Different superscript shows that the quantity is significantly different (*p* < 0.05).

Based on Figure 1, CFS antimicrobial activity produced by *B. subtilis* generated in NB media recorded higher activity for all the pathogens tested in comparison to the CFS from a similar strain but grown in TSB media. Among the three pathogens tested, the maximum recorded antimicrobial activity of CFS from NB was against *P. aeruginosa* at 29.62%, followed by *E. coli* 078:K80 at 7.62%, and the lowest activity at 2.61% from *S. aureus*. Meanwhile, the highest activity recorded for the CFS grown in TSB broth was also against *P. aeruginosa* at 13.93%, while only 0.34% of activity was obtained against *S. aureus*, and no activity was registered when tested against *E. coli* 078:K80. The antimicrobial activity of CFS from NB against *P. aeruginosa* was significantly (*p* < 0.05) high in comparison to the activity of CFS from TSB by 16.32%. The activity of CFS from NB was also higher compared to TSB against the pathogens *E. coli* 078:K80 and *S. aureus* by 7.62 and 1.97%, respectively, but with no significant difference. Hence, the antimicrobial compound released by *B. subtilis* in NB certainly has exhibited better antimicrobial properties compared to the ones produced from TSB.

**Figure 2:**
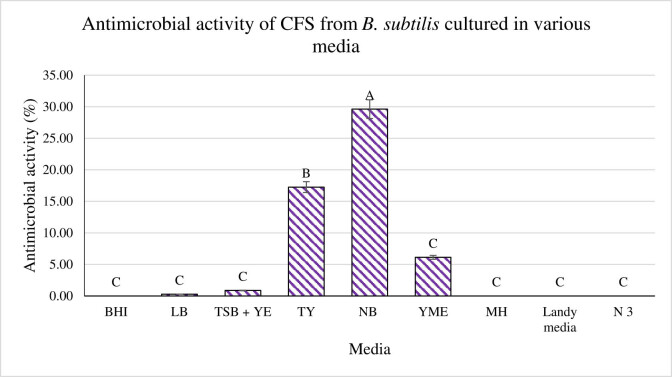
Antimicrobial activity of CFS from *B. subtilis* generated in eight different media (BHI broth, LB broth, [TSB + YE] broth, TY broth, NB broth, YME broth, MH broth, Landy media, and N 3 media). The activity of CFS was tested against *P. aeruginosa*. ^A,B,C^Different superscript shows that the quantity is significantly varied (*p* < 0.05).

Nine different specialized media were prepared with the purpose of producing CFS with higher antimicrobial activity against indicator strain *P. aeruginosa*. Based on the results tabulated in Figure 2, five of the nine media investigated exhibited activity. The highest antimicrobial activity recorded was from CFS produced in NB media at 29.62%, followed by CFS from TY media at 17.26% and YME media at 6.12%. The other two activities recorded were from LB and TSB + YE media, with low activity at 0.28 and 0.87%, respectively. However, no activity was recorded for CFS from BHI, MH, Landy media, and N 3 media. The antimicrobial activity from NB media was high (*p* < 0.05) in comparison to other eight media investigated. Therefore, NB was retained as the growth medium of choice.

**Figure 3:**
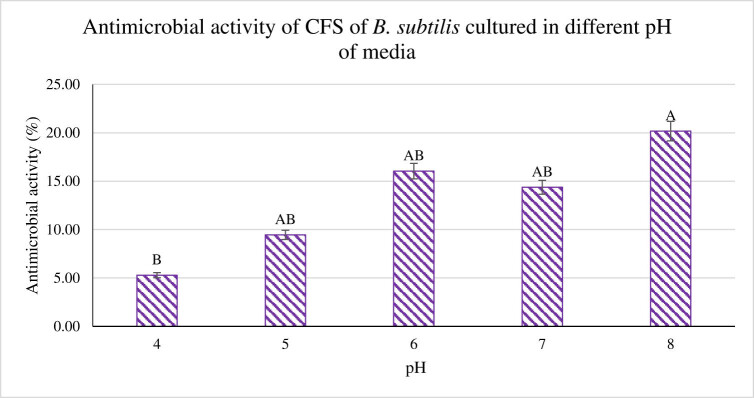
The antimicrobial activity of CFS from *B. subtilis* was tested against *P. aeruginosa* under different pH ranges. ^A,B^Different superscript shows that the quantity is significantly different (*p* < 0.05).

In the present investigation, the pH of media was investigated from pH 4 to pH 8. Based on Figure 3, an increasing trend can be observed. The highest activity was observed at pH 8 at 18.03% and is significantly highest (*p* < 0.05), while the lowest recorded was from pH 4 at 2.73% with a difference margin of 15.3%. The other activities obtained from pH 5, 6, and 7 were 7.01, 13.78, and 12.06%. This investigation highlighted the importance of pH influence on antimicrobial activity.

**Figure 4:**
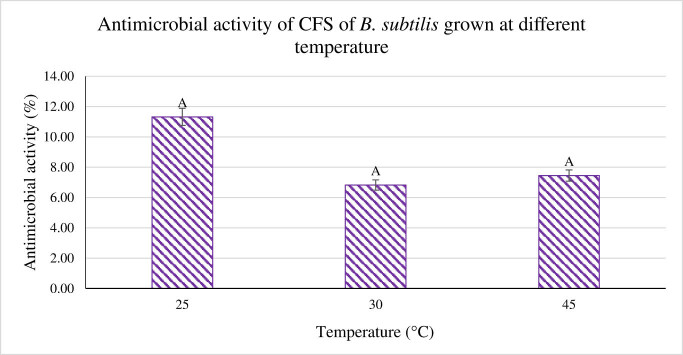
Antimicrobial activity of CFS of *B. subtilis* tested at different temperatures against *P. aeruginosa*. ^A^Superscript shows quantities that are not significantly different (*p* > 0.05).

**Figure 5:**
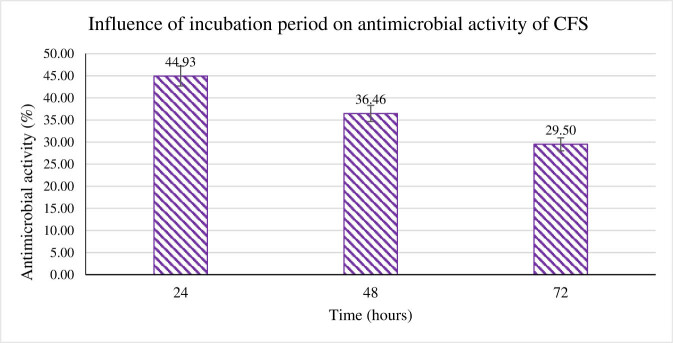
The antimicrobial activity of CFS of *B. subtilis* tested at different incubation periods against *P. aeruginosa*.

The influence of incubation temperature on antimicrobial activity was also studied by incubating growing *B. subtilis* at three different temperatures, i.e., 25, 30, and 45°C. As per the results shown in Figure 4, the inhibitory activity for the three different temperatures was 8.93, 4.31, and 4.95% with no significant difference. Moreover, the incubation period of *B. subtilis* was also monitored. The CFS was extracted from *B. subtilis* every 24 h for a period of 24–72 h. Based on the results tabulated in Figure 5, the antimicrobial activity of CFS toward *P. aeruginosa* scored the highest at 24 h, followed by 48 h with 26.46%, and finally the lowest activity at 72 h with activity of 29.5%. The highest antimicrobial activity was produced after a 24 h incubation period, indicating a clear downward trend in activity. This study concluded that incubating *B. subtilis* longer than 24 h did not influence the antimicrobial activity.

**Figure 6:**
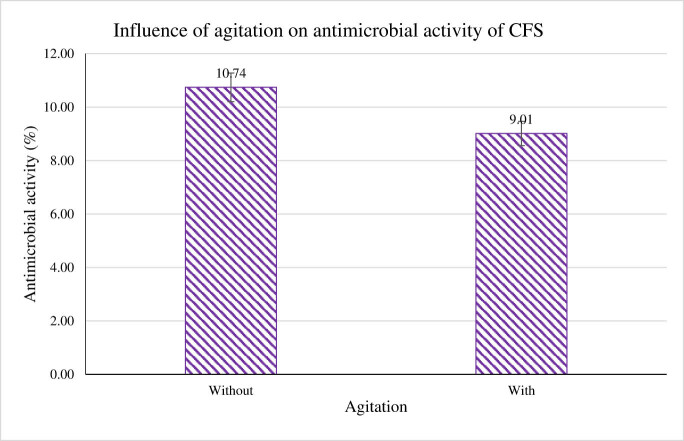
“Without” indicates *B. subtilis* grown without agitation, whereas “With” shows *B. subtilis* culture agitated overnight at 150 rpm. The activity of CFS was tested against *P. aeruginosa*.

The impact of agitation toward the antimicrobial activity of CFS was also observed during the cultivation of *B. subtilis*. Based on the result obtained in Figure 6, CFS antimicrobial activity generated by *B. subtilis* was not influenced by agitation. Culture without agitation scored 10.74% of activity; meanwhile, culture with the presence of agitation scored 1.73% lower antimicrobial activity. Hence, this study proved that agitation is not one of the primary factors governing the antimicrobial activity of the CFS produced. The final investigation carried out was to test the proteinaceous nature of the antimicrobial peptides.

**Figure 7:**
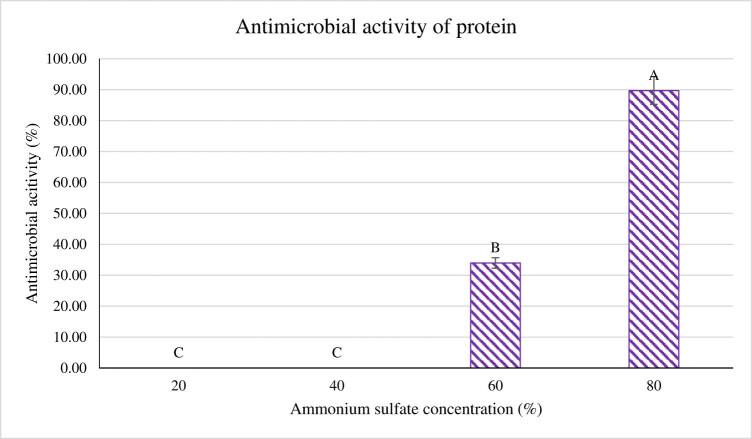
The extracted protein from the CFS at different ammonium sulfate concentrations was tested for its antimicrobial activity against *P. aeruginosa*. ^A,B,C^Different superscript reflects that the quantity is significantly different (*p* < 0.05).

Based on the results in Figure 7, four different ammonium sulfate concentrations were used to carry out this study. The presence of activity was observed at 60 and 80% concentration with the highest recorded at 80%. Meanwhile, no antimicrobial activity was recorded for 20 and 40% ammonium sulfate concentration. The recorded activity for 60 and 80% concentration was 33.96 and 89.72%, respectively. The activity recorded at 89.72% is significantly higher (*p* < 0.05) than the rest of the ammonium sulfate concentrations. The presence of antimicrobial activity of ammonium sulfate proves the proteinaceous nature of CFS.

## 4 Discussion

Antimicrobial resistance is imposing a serious threat to global health. Antimicrobial peptides are rapidly gaining attention for their clinical potential as they have advantages over traditional antibiotics. The antimicrobial peptides synthesized by *B. subtilis* showed a pivotal role in the innate immune system. The antimicrobial activity of CFS from *B. subtilis* had been well documented in various studies. Zhang et al. [29] described the use of CFS produced by *B. subtilis* to effectively reduce biofilm formation of *S. aureus* while reducing its resistance toward antibiotics such as penicillin and gentamicin. The CFS had particularly adjusted the structure of *S. aureus* membrane, making it easily penetrable. Islam et al. [30] also reported the antimicrobial effectiveness of CFS from *B. subtilis* against biofilm production of *P. aeruginosa*. The CFS successfully blocked the biofilm-formation protein (PDB ID: 7M1M) of *P. aeruginosa*, which reduced its biofilm production by 50%.

In this study, among the pathogenic strains tested, the CFS of *B. subtilis* produced the highest antimicrobial activity against *P. aeruginosa*, which is a gram-negative bacteria. Antimicrobial compounds generated by gram-positive bacteria are efficient at targeting the same type of bacteria [31], but the activity of CFS of *B. subtilis* has a broad inhibitory spectrum since it is effective in inhibiting both gram-positive and gram-negative bacteria. Several studies have stated a broad spectrum of *B. subtilis* antibacterial activity, for instance, *B. subtilis* RLID 12.1. Antimicrobial compound generated by particular strain was capable of inhibiting 28 different indicator strains tested, consisting of both gram-positive and gram-negative bacteria and pathogenic yeast [32]. Another similar study by Xie et al. [33] also highlighted the wide spectrum of inhibition of *B. subtilis* LFB112. Bacteriocin produced was able to check 21 different strains, including yeast and gram-positive and gram-negative bacteria. The antimicrobial peptides kill pathogens by either disrupting their membrane or by entering inside bacterial cells to interact with intracellular components.

NB is a better media for antimicrobial compound production among the nine media tested, which is attributed to the composition of the NB media. The higher nitrogen source content, YE, and beef extract facilitated the biosynthesis of antimicrobial compound. Motta and Brandelli [34] also described the importance of source of nitrogen for bacteriocin generation. Bacteriocin synthesis from *Bacillus* sp. P34 strain achieved its maximal activity at 3,200 AU mL^–1^ when cultivated in cheese whey. Khalili Samani et al. [35] described the importance of nitrogen source in the production of bacteriocin. The addition of YE in that particular study elevated the bacteriocin synthesis of *B. subtilis* SB1.

Contrary to most *Bacillus* bacteriocin, this strain operates better at an alkaline pH. The activity recorded was higher in the range from pH 6 to pH 8, with the significantly highest activity achieved at pH 8. Similar findings were reported by Anthony et al. [36]; the bacteriocin production from *Bacillus licheniformis* AnBa9 favored the alkaline pH, and the maximal production was achieved at pH 8. Shayesteh et al. [37] reported that *Bacillus* sp. Sh10 also recorded the highest maximal bacteriocin production at pH 8 when tested between pH 4 and pH 11.

Temperature was the least significant factor in the production of antimicrobial compound. No significant changes were recorded in the activity. However, the highest activity recorded was at 25°C, which was lower than its optimal growth temperature of 37°C. Many studies have observed this similar occurrence, whereby higher bacteriocin activity was recorded at suboptimal temperature [38,39]. The optimal temperature range for bacteriocin production was stated to be in the range between 26 and 37°C [40].

The incubation period profile indicated that the antimicrobial activity was maximal at 24 h and gradually decreased thereafter. Bac-SM01 achieved the highest production at 24 h and stated that incubation time is an important factor in bacteriocin production [41]. Similarly, bacteriocin from *Bacillus* sp. P45 isolated from *Piaractus mesopotamicus* also reported maximal bacteriocin production till 30 h [42]. A longer incubation period leads to the antimicrobial compound adhering to the producer cell surface, thus reducing its efficiency [43].

In this study, agitation did not influence the production of antimicrobial activity. However, contrary to other studies, agitation was impactful in bacteriocin production. Higher subtilosin A activity was recorded when the agitation speed was increased [27]. Hyun et al. [44] also highlighted the importance of agitation in the BLIS BSC35 activity synthesized by *B. subtilis* BSC35. An increasing trend of antimicrobial activity was recorded after an increase in agitation speed.

In this study, 80% ammonium sulfate concentration recorded the highest antimicrobial activity. A few studies also reported similar findings. The BLIS-produced *B. subtilis* BS15 was extracted by 80% saturation of ammonium sulfate [45]. Another study conducted by Cherif et al. [46] also successfully precipitated entomocin 110 generated by *Bacillus thuringiensis* subsp. *Entomocidus* HD110 at 80% saturation. The precipitation of bacteriocin from *B. subtilis* LFB112 was recorded with 80% saturation of ammonium sulfate [33]. Thus, isolated *B. subtilis* showed maximum production of antimicrobial compound, which can be further used for industrial applications [47].

## 5 Conclusions

*B. subtilis* strain can not only act as a biocatalyst in ML-MFC for bioremediation and recovery of energy but is also capable of producing antimicrobial peptide compound. Due to the inhibitory activity of the antimicrobial peptide produced from *B. subtilis* against *P. aeruginosa*, further applications of *B. subtilis* can be explored in the biocontrol of drug-resistant pathogens. The isolated *B. subtilis* was grown best on nutrient media at pH 5 when incubated at 37°C for 1 day and ensured maximal production of antimicrobial compound from *B. subtilis*. An antimicrobial compound is of proteinaceous nature as its nature was confirmed through the precipitation of protein using ammonium sulfate. Hence, findings in this study showcased new values for ML-MFC not just solely for bioremediation and recovery of energy but also due to the presence of high-value antimicrobial compound, which can be further purified for future research.
